# Supplementary material for: All-optical voltage interrogation for probing synaptic plasticity in vivo
Source: Nat Commun. 2025 Oct 3;16:8834. doi: 10.1038/s41467-025-63867-4 (PMC12494759; doi:10.1038/s41467-025-63867-4)
Supplement: Supplementary file 1 — Supplementary Information [file 41467_2025_63867_MOESM1_ESM.pdf]

	JEDI-2P	JEDI-2Psub
**Depolarization (-70 to +30 mV)**		
Tau fast (ms)	0.45±0.08	0.69±0.07
Tau slow (ms)	3.10±0.70	14.9±5.2
Fast component (%)	79.7±6.3	88.5±3.4
**Repolarization (+30 to -70 mV)**		
Tau fast (ms)	1.45±0.10	2.38±0.27
Tau slow (ms)	n/a[a]	19.5±8.0
Fast component (%)	n/a[a]	87.3±5.9

Supplementary Table 1. Comparison of kinetics between JEDI-2P and JEDI-2Psub. JEDI-2Psub has slower off-kinetics compared with JEDI-2P. Depolarization was performed from −70 mV to +30 mV, while repolarization was performed from +30 mV back to –70 mV. Values represent means from n=10 HEK293A cells for all GEVIs at 32°–33°C. Cells were illuminated with 475 nm light and emitted photons recorded with a photomultiplier tube (PMT) at 80 kHz. [a]These kinetics were best fit by a double-exponential function where one exponential describes the kinetics and the second describes a slow photobleaching component (not shown).

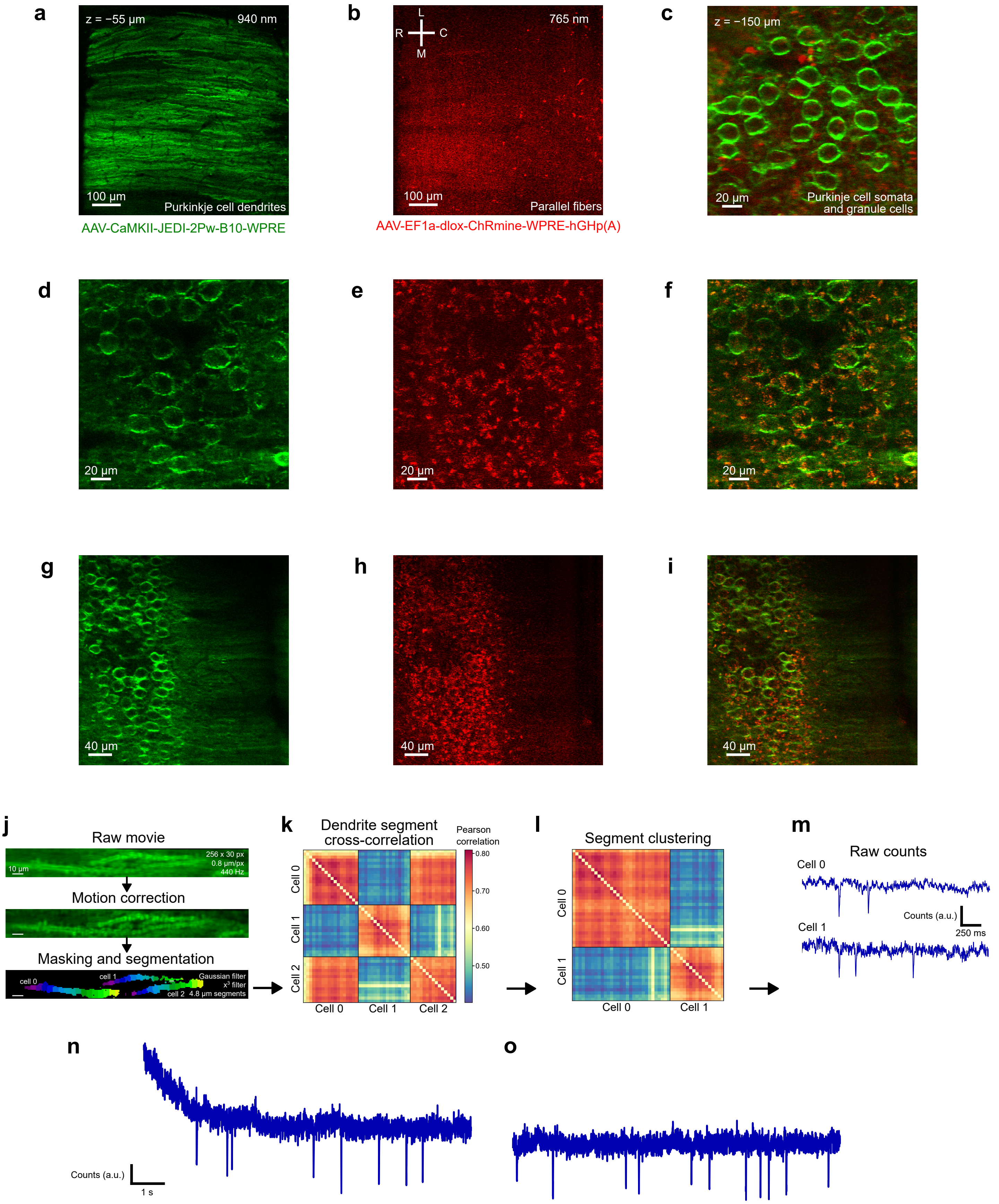

Supplementary Figure 1. Co-expression strategy and data processing pipeline. (**a**) JEDI-2Psub is expressed selectively in Purkinje cell dendrites (shown) and somata (image captured at 940 nm, 55 µm below the pia) and (**b**) ChRMine is expressed in granule cells and parallel fibers (image captured at 765 nm) to excite mScarlet. (**c**) An image of co-expression in the Purkinje cell layer, 150 µm below the pia, showing Purkinje cell soma (green) and granule cells (red). (**d-f**) Images showing co-expression of JEDI-2Psub (green) and ChRMine (red) in a single field-of-view, alongside the composite image. (**g-i**) Images showing co-expression of JEDI-2Psub (green) and ChRMine (red) in a different animal from (**d-f**) , alongside the composite image. (**j**) Recordings are processed by first motion correcting them using Suite2p and then spatially filtering and segmenting Purkinje cell dendrites into 4.8 µm sections. (**k**) The raw activity from each segment is subsequently correlated with every other segment, giving a cross correlation matrix. (**l**) This cross correlation matrix is then clustered to identify putative dendrites that belong to the same cell. (**m**) Resulting raw traces, with JEDI-2Psub going from bright at baseline to dim during depolarization. (**n**) The first 10 s of raw trace from a Purkinje cell dendrite compared to (**o**) the final 10 s of a three minute recording. Count axes are aligned between the two plots.

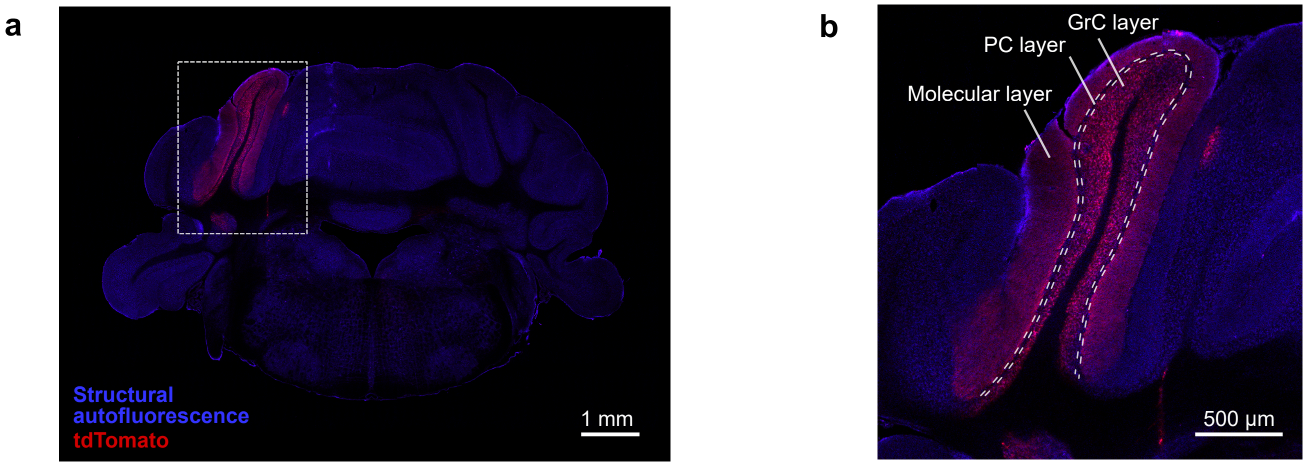

Supplementary Figure 2. Histological validation of opsin expression in granule cells. (**a**) Coronal section of the cerebellum of a Math1-Cre mouse injected with Cre-dependent ChR2-tdTomato virus in lobule simplex. The ChR2-tdTomato fusion protein (red) was restricted to the injected cerebellar lobule and not present in any pre-cerebellar nuclei (structural autofluorescence is shown in blue). (**b**) Zoom in of the highlighted region in panel **a** showing tdTomato fluorescent labelling in granule cell (GrC) layer (granule cell somata) and molecular layer (parallel fiber axons).

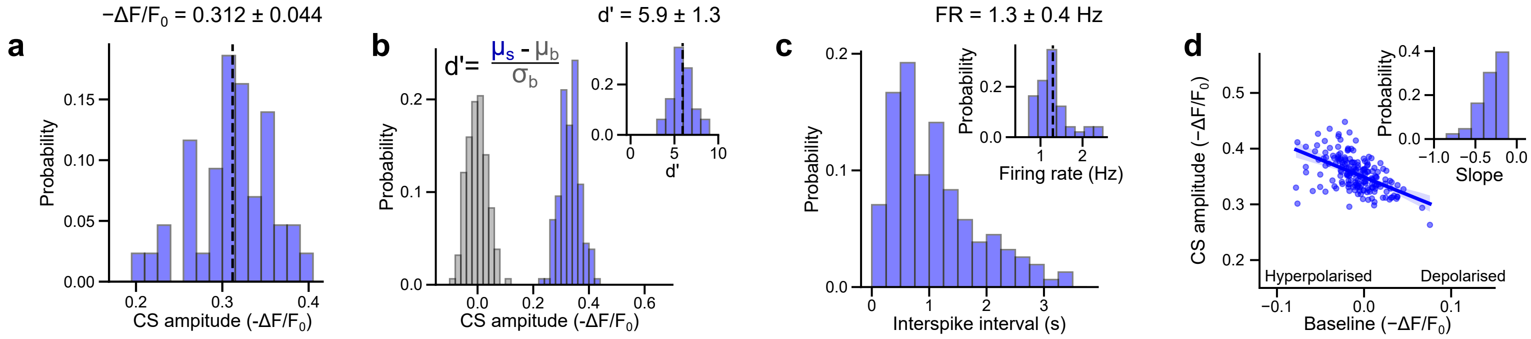

Supplementary Figure 3. 2-photon voltage imaging of Purkinje cell spontaneous complex spike signals. (**a**) A histogram of mean complex spike signal amplitudes across all cells (n=43 cells across N=4 mice). (**b**) A histogram of discriminability index *d′* for all spontaneous complex spike signals for a representative PC (blue) against baseline (grey), inset: a histogram of *d′* across all cells. (**c**) Interspike interval measured across a single PC, inset: firing rate (FR) measured across all PCs. (**d**) A scatter plot of complex spike amplitudes (baseline to peak) against baselines, for n=181 spikes in a single cell (ρ=-0.523, p=4.18 x 10^{-14}, fitted by linear regression and tested via a two-sided Wald test, shaded area represents 95% confidence interval). Inset: A histogram of gradients for n=43 cells and N=4 mice with a mean ± std=-0.299 ± 0.163.

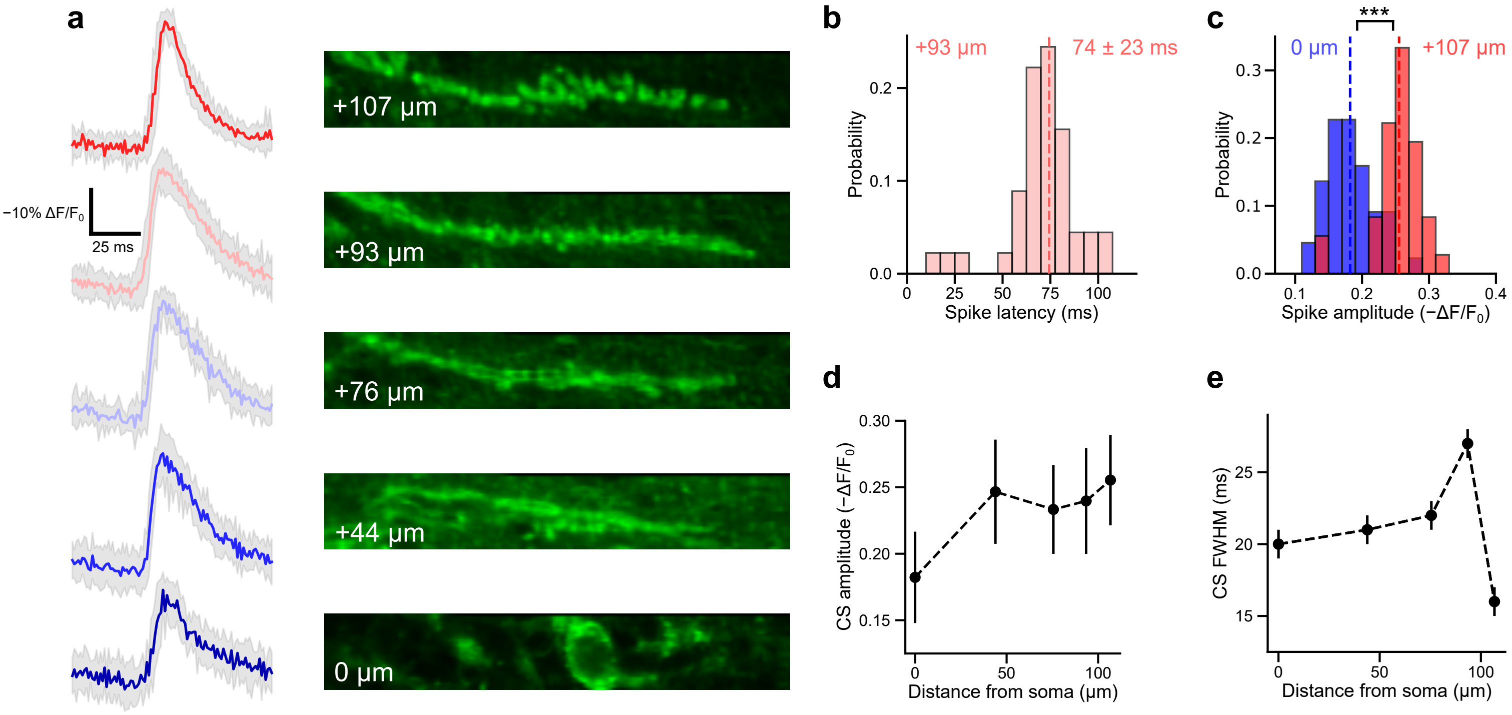

Supplementary Figure 4. Spatial analysis of evoked dendritic responses. (**a**) The sensory response (left) of the same neuron (right) is measured at multiple distances from the soma. Each solid line is the 1 kHz upsampled average of n=58 trials, with the shaded line representing ± 1 std. (**b**) Histogram of complex spike (CS) latencies at a distance of 93 µm from the soma, N=45 CSs. (**c**) Comparing the amplitude of somatic CSs (n=44 spikes, amp=0.182 ± 0.034) and distal CSs (n=36, amp=0.255 ± 0.034). two-sided Mann-Whitney U-test $p=1.01 \times 10^{-10}$. (**d**) Plot of evoked complex spike amplitude vs distance from the soma. Error bars, mean ± 1 std. (**e**) Plot of evoked complex spike FWHM vs distance from the soma. Error bars, mean ± 1 std.

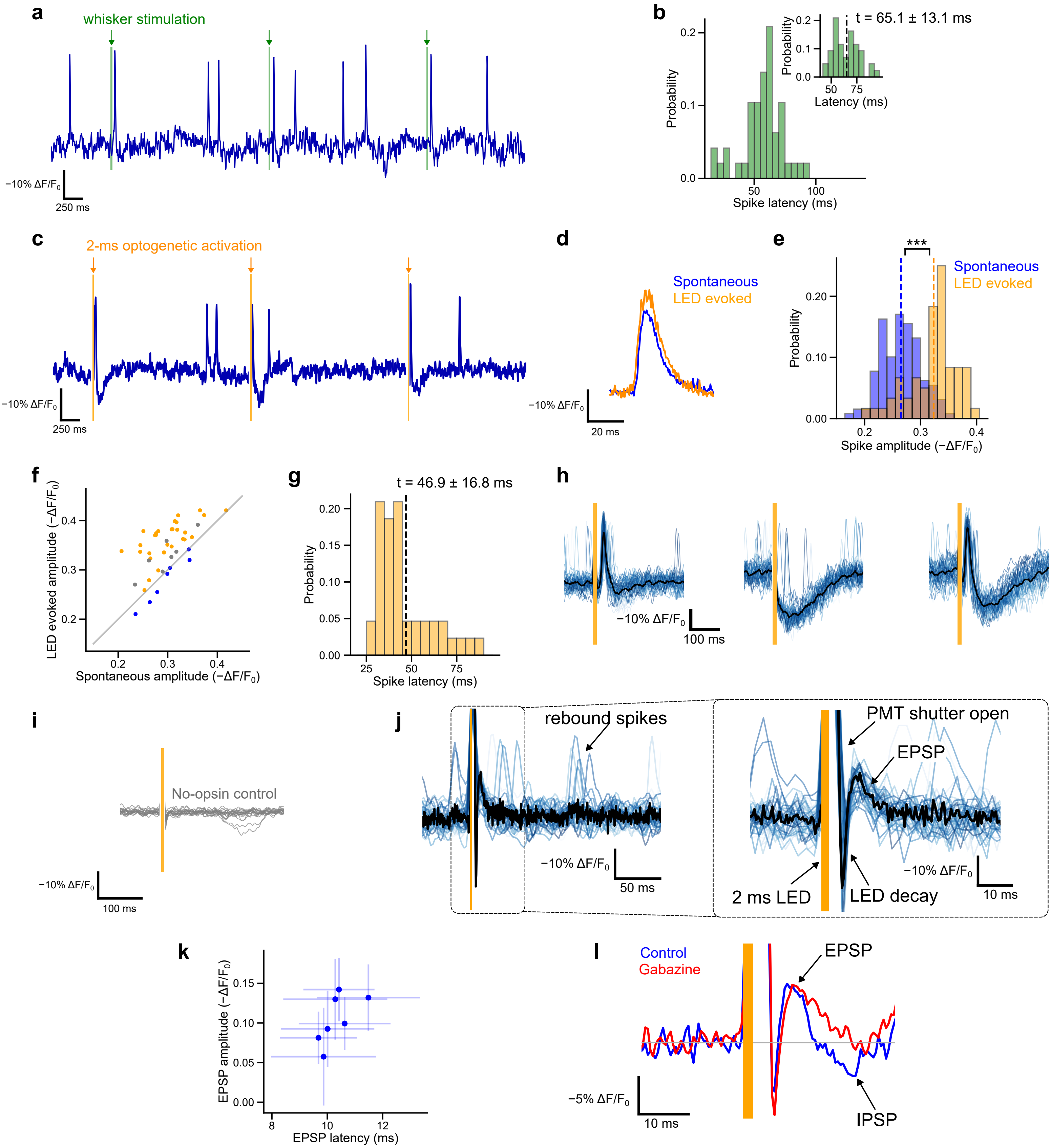

Supplementary Figure 5. Sensory-evoked and optically-evoked dendritic responses. (**a**) 6 s trace of a PC during sensory stimulation (1-frame Gaussian filter). A 25 ms air puff is applied to the whisker pad of the mouse at 0.5 Hz. (**b**) For the same cell, a histogram of spike latencies of sensory evoked spikes (n=48 spikes, t=57.5 ± 20.0 ms). Inset: average over n=43 cells and N=4 animals. (**c**) 6 s trace of optogenetic activation of GrCs with 2 ms widefield LED illumination at 0.5 Hz. For certain cells, optogenetic activation of GrCs evokes a large excitatory response with an average amplitude that is larger than that of spontaneous complex spikes. (**d**) Mean spike waveform for the cell shown in **c**. (**e**) For the same cell the histogram of all spontaneous and LED evoked amplitudes are shown. (**f**) Across all responsive cells, the average evoked amplitude is larger than spontaneous complex spike signals, Wilcoxon signed-rank test p=3.57 x 10^{-8}. (**g**) A histogram of the latency of optogenetically evoked responses across all responsive cells. (**h**) There is a diversity of optogenetically evoked responses, the overlay of 60 trials for three different cells, black line represents the mean response. (**i**) Optical response without an opsin being co-expressed, each trace is the average of 60 trials in one PC, and n=19 cells shown across N=2 mice. (**j**) Example of a PC with a clear EPSP in response to optogenetic activation of GrCs. (**k**) The latency and amplitude of all cells displaying clear optically induced EPSPs, amplitude=−10.5 ± 2.9% $\Delta F/F_0$, latency=10.3±0.6 ms (mean ± std, n=7 cells). (**l**) Change in EPSP before (blue) and after (red) gabazine. Grey line represents $\Delta F/F_0$=0%.

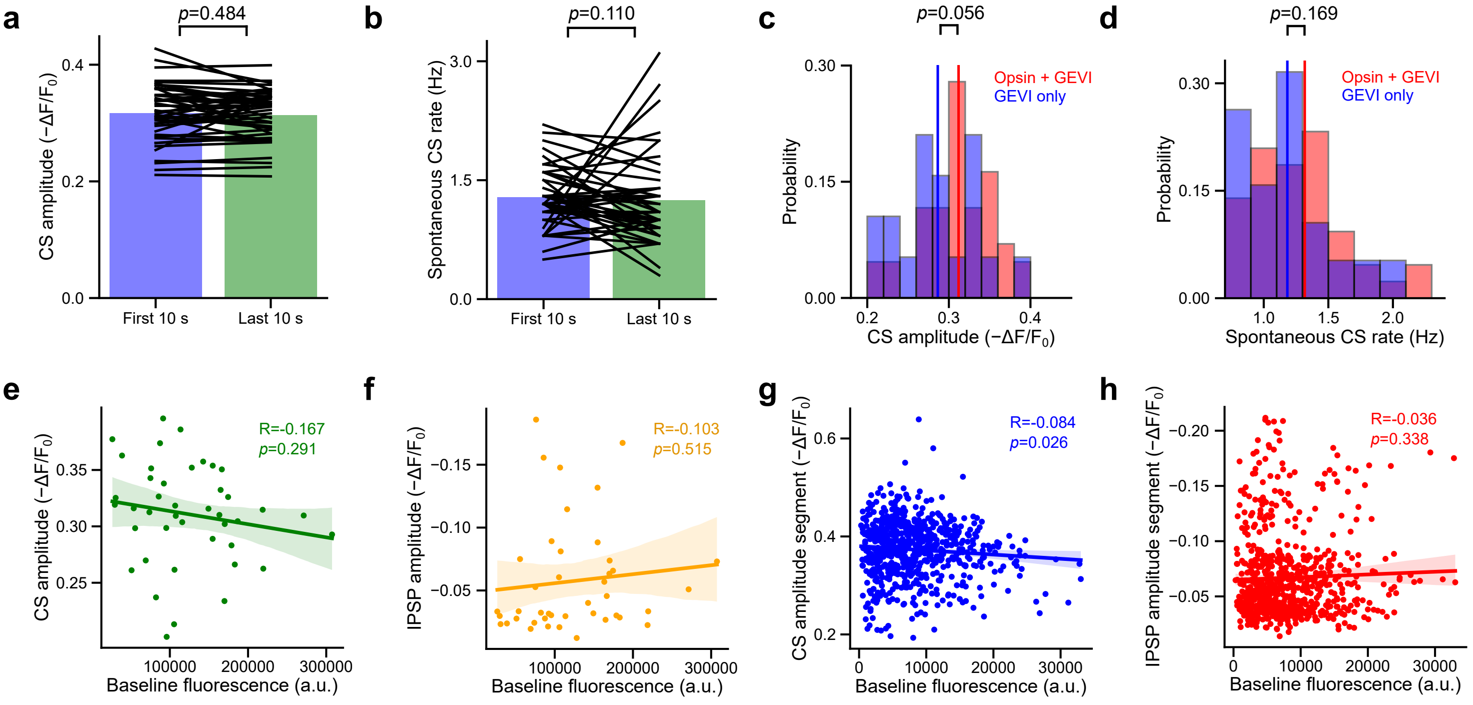

Supplementary Figure 6. Voltage imaging and cross-talk controls. (**a**) A comparison of complex spike amplitude in the first 10 s and the last 10 s of the recording (n = 43 cells, mean start amplitude -0.32 ± 0.05 $\Delta F/F_0$, mean end amplitude -0.31 ± 0.04 $\Delta F/F_0$, tested via a Wilcoxon Signed-Rank Test). (**b**) A comparison of complex spike rate in the first 10 s and the last 10 s of the recording (n = 43 cells, mean start rate 1.3 ± 0.4 Hz, mean end rate 1.2 ± 0.6 Hz, tested via a Wilcoxon Signed-Rank Test). (**c**) A histogram of complex spike amplitude in neurons of mice co-expressing an opsin and GEVI (red, mean amp = -0.31 ± 0.04 $\Delta F/F_0$, n = 43 cells) and GEVI-only mice (blue, mean amp = -0.29 ± 0.05 $\Delta F/F_0$, n = 19 cells), tested via a Mann-Whitney U Test. (**d**) A histogram of complex spike rate in neurons of mice co-expressing an opsin and GEVI (red, mean rate = 1.3 ± 0.4 Hz, n = 43 cells) and GEVI-only mice (blue, mean rate = 1.2 ± 0.3 Hz, n =19 cells), tested via a Mann-Whitney U Test. (**e**) Baseline fluorescence against complex spike amplitude (n = 42 cells). (**f**) Baseline fluorescence against IPSP amplitude (n = 42 cells). (**g**) Baseline fluorescence against complex spike amplitude for dendritic segments (n = 714 segments). (**h**) Baseline fluorescence against complex IPSP amplitude for dendritic segments (n = 714 segments). All plots are fitted by linear regression and tested via a two-sided Wald test. Shaded area represents 95% confidence interval.

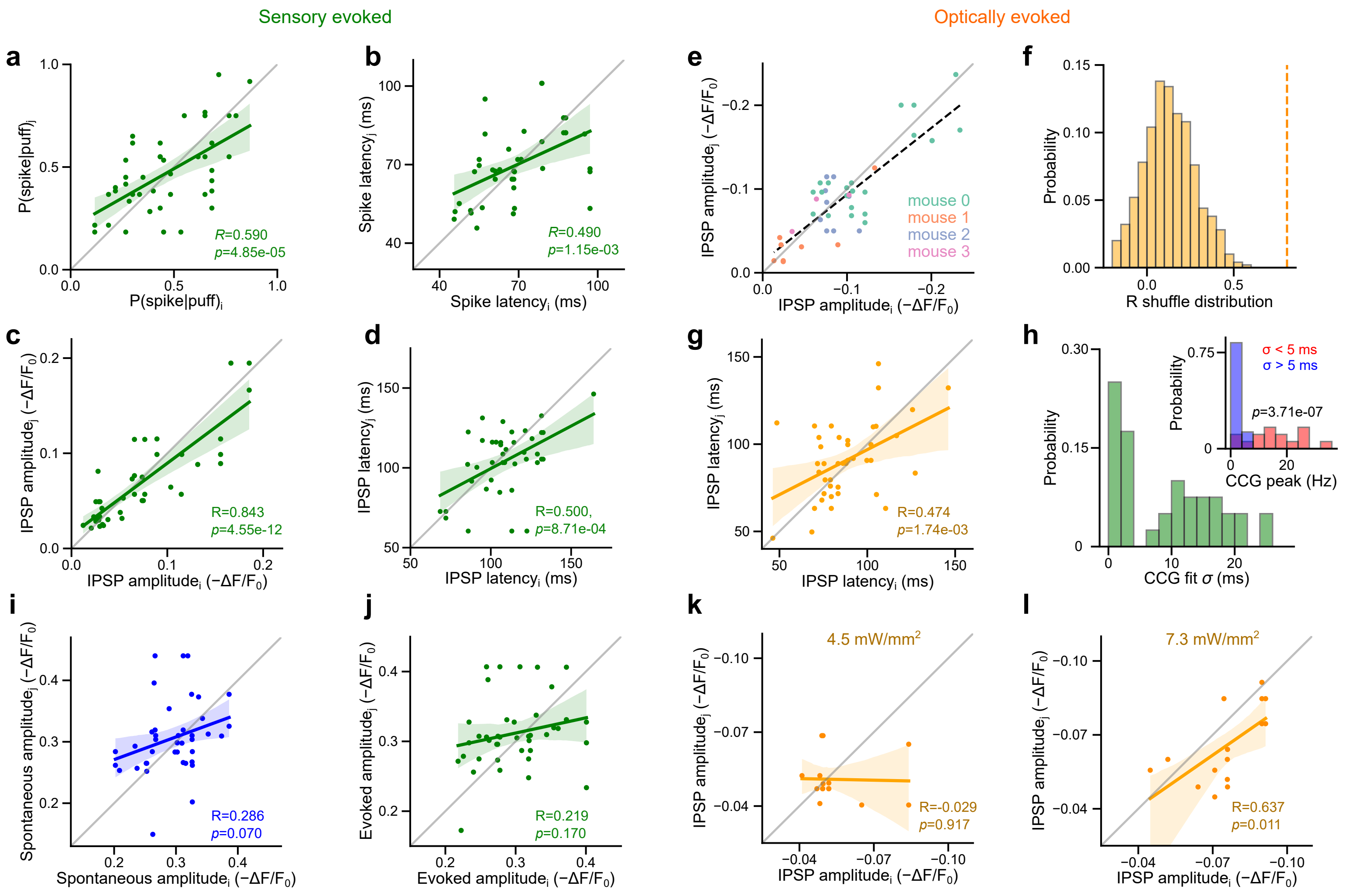

Supplementary Figure 7. Correlation in voltage signals between neighbouring PCs. (**a**) For all pairs of PCs *(i,j)* in the same field of view (FOV), the probability of a sensory evoked complex spike event of PC_i is plotted against PC_j. (**b**) For the same pairs of PCs, the mean latency of all sensory evoked complex spike events is plotted. (**c**) The mean amplitude of all sensory evoked IPSP events is plotted for all pairs of PCs in the same FOV as is (**d**) the mean latency. (**e**) The mean optically evoked IPSP amplitude between all pairs of PCs as shown in Fig. 2, but broken down by mouse. (**f**) A distribution of the correlation coefficient from 500 shuffles, between random pairs of cells that are not in the same FOV but are from the same mouse, the measured correlation coefficient (yellow dashed line) lies outside the 99th percentile indicating the correlation is not due to within mouse correlations. (**g**) The mean optically evoked IPSP latency between all pairs of PCs in the same FOV. (**h**) The cross-correlogram (CCG) between detected complex spike events in the same FOV is fitted to a Gaussian and a histogram of the standard deviation σ of those fits is plotted. There is a clear bimodal distribution around $\sigma < 5$ ms. Pairs of PCs with $\sigma < 5$ ms have a significantly greater CCG peak (inset, red) than $\sigma > 5$ ms (inset, blue), Mann-Whitney U Test, indicating PC pairs with fits $\sigma < 5$ ms are likely from the same microzone. (**i**) The mean spontaneous complex spike amplitude of neighbouring PCs was not significantly correlated, nor was (**j**) the sensory evoked amplitude. (**k, l**) The correlation between optically evoked IPSPs amplitude increased with optical intensity (n=13 cells across one mouse). All plots are fitted by linear regression and tested via a two-sided Wald test. Shaded area represents 95% confidence interval.

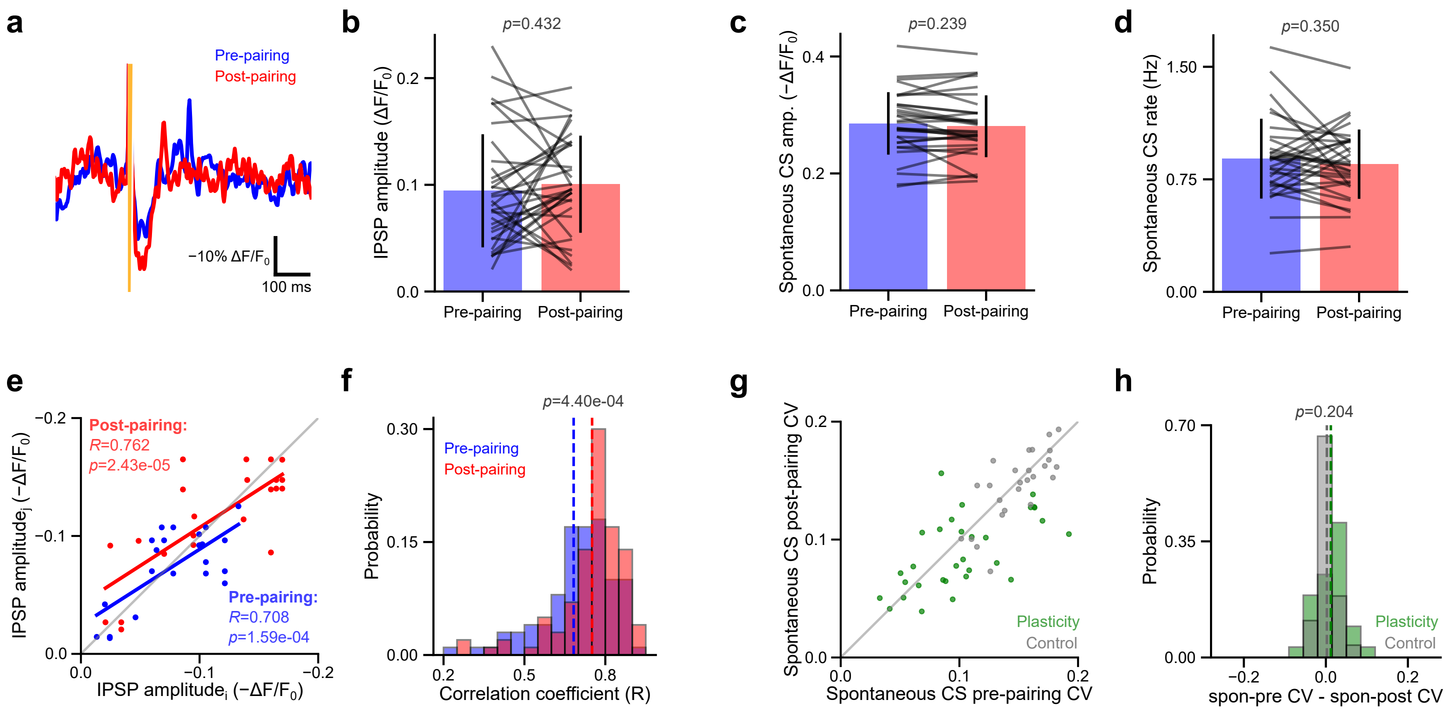

Supplementary Figure 8. Plasticity controls. (**a**) Overlay of two single trials pre- and post-pairing of the cell shown in Fig. 4b. (**b**) Comparing the IPSP amplitude pre- (blue) and post-pairing (red) when the granule cell and climbing fiber stimuli are reversed, such that the air puff occurs 80 ms after the LED stimulation (Wilcoxon Signed-Rank test, p=0.432, n=32 cells across N=4 mice). (**c**) A comparison of mean spontaneous complex spike (CS) amplitude pre- and post-pairing showing no significant difference (Wilcoxon Signed-Rank test, p=0.239, n=32 cells across N=4 mice). (**d**) A comparison of mean spontaneous CS spike rate pre- and post-pairing showing no significant difference (Wilcoxon Signed-Rank test, p=0.350). (**e**) Correlation in mean IPSP amplitude between pairs of PCs in the same FOV. The correlation in mean IPSP amplitude is marginally stronger in post-pairing compared with pre-pairing, (**f**) which is then tested via bootstrapping by randomly holding out 50% of the data for 100 trials and testing the resultant distribution of correlation coefficients with a Mann-Whitney U-test ($p=4.40 \times 10^{-4}$). (**g**) Coefficient of variation (CV) for spontaneous CS amplitudes plotted pre- and post-pairing, for both the plasticity condition (green) and control condition (grey). (**h**) There is no significant difference between the two conditions indicating that the distribution of spontaneous CS signals does not vary under the pairing protocol.

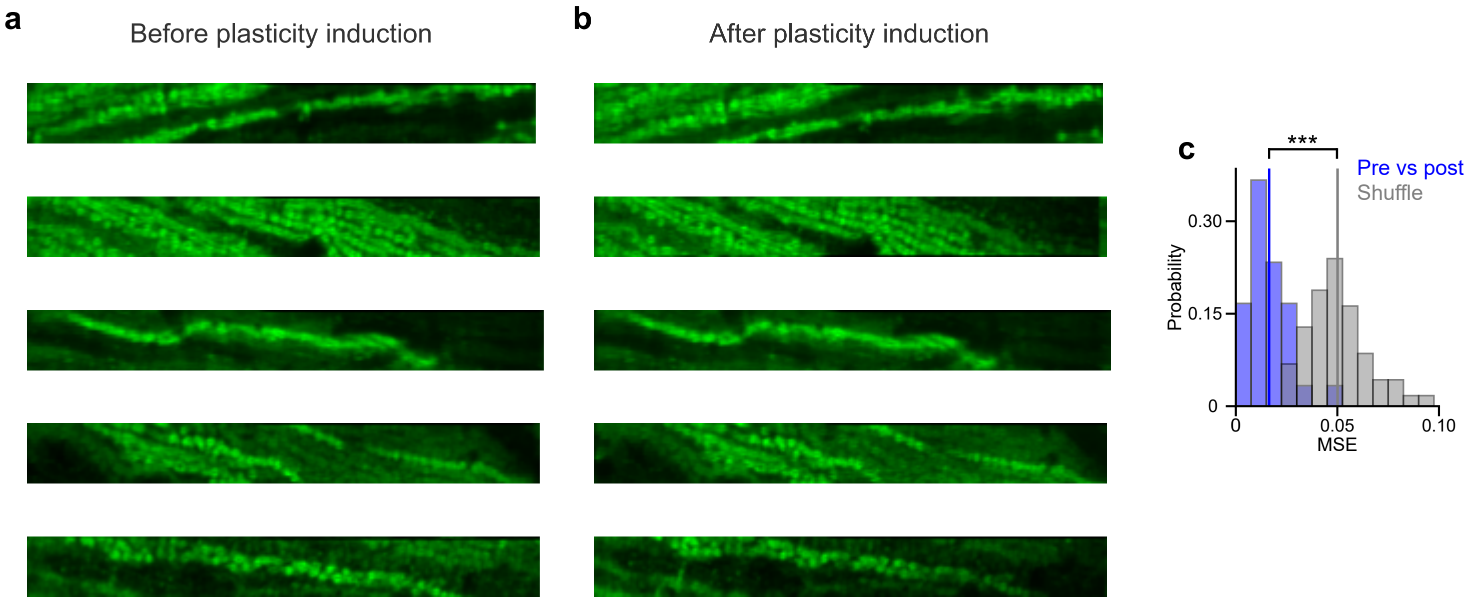

Supplementary Figure 9. Comparing fields of view before and after plasticity induction. (**a**) Five representative fields of view before plasticity induction and (**b**) the same fields of view after plasticity induction. (**c**) The mean squared error between the same pairs of fields of view before and after plasticity induction is 0.016 ± 0.009 (mean ± std across n=30 FOVs), which is significantly smaller than a shuffle distribution of MSE between different fields of view (MSE=0.050 ± 0.015, n=117 shuffles, Mann-Whitney U Test $p=5.66 \times 10^{-16}$).
